# Treatment of ovarian endometriosis: Number 1 – 2026

**DOI:** 10.61622/rbgo/2026FPS1

**Published:** 2026-02-12

**Authors:** Marcos Tcherniakovsky, Márcia Mendonça Carneiro, João Sabino Lahorgue da Cunha, Carlos Alberto Petta, Carlos Augusto Pires Costa Lino, Corival Lisboa Alves de Castro, Eduardo Schor, João Nogueira, Marco Aurélio Pinho de Oliveira, Maurício Simões Abrão, Omero Benedicto Poli, Ricardo de Almeida Quintairos, Sidney Pearce, Helizabet Salomão Abdalla, Sergio Podgaec, Julio Cesar Rosa e Silva

**Affiliations:** 1 Setor de Videoendoscopia Ginecológica e Endometriose Faculdade de Medicina do ABC Santo André SP Brazil Setor de Videoendoscopia Ginecológica e Endometriose, Faculdade de Medicina do ABC, Santo André, SP, Brazil.; 2 Universidade Federal de Minas Gerais Faculdade de Medicina Departamento de Ginecologia e Obstetrícia Belo Horizonte MG Brazil Departamento de Ginecologia e Obstetrícia, Faculdade de Medicina, Universidade Federal de Minas Gerais, Belo Horizonte, MG, Brazil.; 3 Universidade Federal do Rio Grande do Sul Departamento de Ginecologia e Obstetrícia Porto Alegre RS Brazil Departamento de Ginecologia e Obstetrícia, Universidade Federal do Rio Grande do Sul, Porto Alegre, RS, Brazil.; 4 Universidade Estadual de Campinas Campinas SP Brazil Universidade Estadual de Campinas, Campinas, SP, Brazil.; 5 Clínica Fertilidade & Vida Campinas SP Brazil Clínica Fertilidade & Vida, Campinas, SP, Brazil.; 6 Hospital Sírio-Libanês Serviço de Reprodução Assistida São Paulo SP Brazil Serviço de Reprodução Assistida, Hospital Sírio-Libanês, São Paulo, SP, Brazil.; 7 Hospital Aliança Salvador BA Brazil Hospital Aliança, Salvador, BA, Brazil.; 8 Instituto de Perinatologia da Bahia Salvador BA Brazil Instituto de Perinatologia da Bahia, Salvador, BA, Brazil.; 9 Hospital Geral de Goiânia Goiânia GO Brazil Hospital Geral de Goiânia, Goiânia, GO, Brazil.; 10 Universidade Federal de São Paulo Escola Paulista de Medicina São Paulo SP Brazil Escola Paulista de Medicina, Universidade Federal de São Paulo, São Paulo, SP, Brazil.; 11 Sociedade Brazileira de Endometriose e Cirurgia Minimamente Invasiva São Paulo SP Brazil Sociedade Brazileira de Endometriose e Cirurgia Minimamente Invasiva, São Paulo, SP, Brazil.; 12 Universidade Federal do Maranhão São Luís MA Brazil Universidade Federal do Maranhão, São Luís, MA, Brazil.; 13 Universidade do Estado do Rio de Janeiro Faculdade de Ciências Médicas Rio de Janeiro RJ Brazil Faculdade de Ciências Médicas, Universidade do Estado do Rio de Janeiro, Rio de Janeiro, RJ, Brazil.; 14 Hospital Beneficência Portuguesa de São Paulo Divisão de Ginecologia São Paulo SP Brazil Divisão de Ginecologia, Hospital Beneficência Portuguesa de São Paulo, São Paulo, SP, Brazil.; 15 Universidade de São Paulo Faculdade de Medicina Departamento de Ginecologia e Obstetrícia São Paulo SP Brazil Departamento de Ginecologia e Obstetrícia, Faculdade de Medicina, Universidade de São Paulo, São Paulo, SP, Brazil.; 16 Universidade de São Paulo Faculdade de Medicina de Ribeirão Preto Departamento de Ginecologia e Obstetrícia Ribeirão Preto SP Brazil Departamento de Ginecologia e Obstetrícia, Faculdade de Medicina de Ribeirão Preto, Universidade de São Paulo, Ribeirão Preto, SP, Brazil.; 17 Hospital Porto Dias Núcleo de Endometriose Belém PA Brazil Núcleo de Endometriose, Hospital Porto Dias, Belém, PA, Brazil.; 18 Centro Universitário Christus Fortaleza CE Brazil Centro Universitário Christus, Fortaleza, CE, Brazil.; 19 Santa Casa de São Paulo Faculdade de Ciências Médicas São Paulo SP Brazil Faculdade de Ciências Médicas, Santa Casa de São Paulo, São Paulo, SP, Brazil.; 20 Universidade de São Paulo Faculdade de Medicina Departamento de Ginecologia e Obstetrícia São Paulo SP Brazil Departamento de Ginecologia e Obstetrícia, Faculdade de Medicina, Universidade de São Paulo, São Paulo, SP, Brazil.; 21 Sociedade Beneficente Israelita Brazileira Albert Einstein São Paulo SP Brazil Sociedade Beneficente Israelita Brazileira Albert Einstein, São Paulo, SP, Brazil.; 22 Universidade de São Paulo Faculdade de Medicina de Ribeirão Preto Departamento de Ginecologia e Obstetrícia Ribeirão Preto SP Brazil Departamento de Ginecologia e Obstetrícia, Faculdade de Medicina de Ribeirão Preto, Universidade de São Paulo, Ribeirão Preto, SP, Brazil.

## Key points

- Endometriosis is a common benign disease that can compromise women's quality of life and their fertility.
- Ovarian endometrioma is a form of the disease that can compromise women's fertility and cause pain and discomfort.
- Fertility preservation is a key point to consider in the care of women with endometriosis, especially those with ovarian endometriomas.
- The etiopathogenesis of the disease and the development of ovarian endometrioma are not yet fully understood.
- Several mechanisms are responsible for infertility in women with endometriosis, including increased production of cytokines and chemokines, an altered hormonal environment, increased oxidative stress, and impaired tubal and sperm function. Furthermore, endometriomas can interfere with folliculogenesis.

## Recommendations

- Surgical treatment of endometriomas, when indicated, should preferably be performed with drainage and excision of the pseudocapsule.
- Alternative techniques that better preserve ovarian reserve, such as drainage and sclerotherapy or alcohol ablation, may be performed selected cases, provided that this is agreed upon with the patient.
- Fertility preservation should be an important point to consider in the care of women with endometriosis, especially those with ovarian endometriomas.
- Women with unoperated bilateral endometriomas and those who have previously had unilateral endometriomas removed and require surgery for a contralateral recurrence should be advised about fertility preservation and available strategies, including cryopreservation of embryos and oocytes.
- As age is the most important prognostic factor, all patients should be aware of the effect of age on their fertility plans.
- Women should be individually advised about the risks, benefits, and costs involved in disease treatment, including medical and surgical treatment and assisted reproduction.
- Care by a multidisciplinary team with expertise in endometriosis is fundamental to achieving better results.

## Background

An ovarian endometrioma is best defined as a pseudocyst originating from ectopic endometrial tissue within the ovary that progressively invaginates the ovarian cortex.^(1)^ It contains a thick, brown fluid, sometimes referred to as a "chocolate cyst" composed of metabolized blood and endometrial cells. Endometriosis is often difficult to diagnose, particularly in asymptomatic women. The literature shows a wide variation in the incidence of endometriosis in this group, which is estimated to be between 2% and 50%. Among asymptomatic women diagnosed with endometriosis, 17% to 44% are reported to have endometriomas.^(2)^ However, the precise incidence of asymptomatic endometriomas is unknown, largely because many of these women do not seek medical treatment.

Studies demonstrate a significant association between endometrioma and deep or infiltrative endometriosis in locations other than the ovary with painful symptoms that are not solely related to the presence of the endometrioma.^(3)^

Another important characteristic of endometriomas is that, in many cases, there is no correlation between the stage of endometriosis and the severity of symptoms.^(4)^

The diagnosis of ovarian endometrioma may be suggested by the patient's clinical complaint (dysmenorrhea, chronic pelvic pain, infertility) and/or suspected by physical examination, but should always be confirmed by transvaginal ultrasound (US) or pelvic magnetic resonance imaging (MRI). Confirmation is achieved through laparoscopic inspection and histopathological confirmation is the gold standard.

With advances in imaging techniques, both pelvic US and pelvic MRI can be used in the diagnosis of endometriomas. Ultrasound performed by professionals with expertise has shown good specificity (96%) and sensitivity (93%) and MRI provides similar results to those of US.^(5)^

Treatment options include expectant management and medical or surgical therapy, depending on the clinical picture in relation to the intensity of symptoms and severity of the disease, the desire for pregnancy, the patient's age, the size of the endometrioma, and a history of surgery for the treatment of endometriosis, especially ovarian endometriosis.^(6)^

Although there is no consensus on the best treatment for ovarian endometriomas, whether clinical or surgical, due to the ineffectiveness of complete involution of these cysts with clinical treatment, surgery often becomes necessary. On the other hand, surgery carries a significantly higher risk of ovarian tissue damage and, consequently, a reduction in ovarian reserve and impairment of future reproductive potential.^(6)^

## Asymptomatic endometriomas: como conduzir?

The criteria for surgical intervention in cases of women with asymptomatic endometriomas are based on suspected malignancy (US, MRI, tumor markers), women over 40 years of age, large endometriomas, or if significant growth is observed. Surgical intervention would not be appropriate in young women and/or those with small endometriomas.^(7)^ Another important detail is that surgical intervention would also provide a definitive histopathological diagnosis and prevent possible cyst rupture, which rarely occurs, thus protecting the patient from emergency surgery.

Although conservative treatment should include clinical and imaging follow-up, the follow-up time and interval are unknown. Treatment should be individualized, based on previous and current treatments and the severity of symptoms and disease.^(7)^

There is no consensus in the literature on how these women should be monitored and how frequently. Individualized assessment is recommended, taking into account the intensity of symptoms, previous treatments, and reproductive desires.^(8)^

A cystectomy (stripping technique) should be performed for endometrioma removal, as indicated by the European Society of Human Reproduction and Embryology (ESHRE), despite the lack of consensus in the literature.^(8)^ Puncture and aspiration of the entire contents appears to be ineffective as a treatment method and is associated with a high recurrence rate. Drainage followed by electrocoagulation of the cyst has also proven less effective than complete excision of the cyst capsule.^(9)^

## Endometriomas and infertility: excision or not? What is the best approach to avoid compromising fertility?

The evaluation of endometriomas in infertile patients has attracted increasing interest due to the lack of consensus regarding the causal relationship between their presence and infertility. On the other hand, several articles demonstrate the loss of follicular stock, leading to a loss of ovarian reserve, especially in cases of bilateral endometriomas.^(10,11)^ Garcia-Velasco and Somigliana^(12)^ related specific situations regarding the possible approach to endometriomas in women candidates for assisted reproductive techniques. Situations in which surgery with removal of the capsule would be beneficial (in favor) or detrimental (against) are listed in chart 1.

**Chart 1:** Considerations in the approach to endometrioma in women candidates for in vitro fertilization

	In favor	Against
Previous endometriosis surgery	No	Yes
Ovarian reserve	Normal	Changed
Symptoms	Yes	No
Ovarian involvement	Unilateral	Bilateral
Growth	Yes	Stable
Suspicion of malignancy on US	No	Yes

Source: Adapted from Garcia-Velasco and Somigliana (2009).^(12)^

Women with endometrioma should be advised about the risks of reduced ovarian function after surgery and the possible loss of the ovary. The decision to proceed with surgery must be shared and carefully considered, especially if the woman has undergone previous ovarian surgery.^(9)^ In these cases, the woman should receive guidance on fertility preservation through egg freezing.^(13)^

Ovarian responsiveness in in vitro fertilization (IVF) cycles is not affected by the presence of unoperated endometriomas, suggesting these lesions do not significantly impair ovarian reserve. In endometriomas smaller than 4 cm, the pregnancy rate is similar in patients undergoing endometrioma excision compared to non-operated patients. Therefore, surgery should be avoided in these cases, mainly to preserve ovarian follicles and, consequently, ovarian reserve.^(14)^ In cases of bilateral endometriomas, the criteria for surgical intervention should be even more restrictive, as the risks of ovarian failure can reach up to 2.4%.^(10)^ There is no recommendation for performing endometrioma surgery with the aim of improving IVF success rates.^(8)^

## Endometriomas and pelvic pain: when and how to treat them?

The relationship between chronic pelvic pain and endometriomas is not very well established.^(15)^ In most cases, the symptoms cannot be solely related to endometriomas, periovarian adhesions, or deeply infiltrating endometriosis.^(2)^

Even though the reduction in the size of endometriomas with hormonal treatment varies from 14% to 89%, average of 51%, there are no studies correlating hormonal suppression with chronic pelvic pain in women with endometriomas.^(16,17)^

Surgical treatment of symptomatic endometriomas appears to be the first option in women with completed families.^(7)^ It includes drainage with or without sclerotherapy, drainage with diathermy or laser with vaporization of the cyst wall, drainage and cystectomy (stripping technique), and finally, radical treatment in the form of hysterectomy with or without oophorectomy.

Although uncontrolled studies have shown symptom relief in a large proportion of patients undergoing surgery for endometrioma,^(17)^ a high recurrence rate has been observed in these cases.^(7)^

Endometrioma drainage by laparoscopy or US is associated with a high and rapid recurrence rate and is rarely effective in relieving symptoms.^(18,19)^ It may be associated with a higher risk of infection and pelvic adhesions.^(20)^

Endometrioma excision involves drainage followed by excision of the cyst pseudocapsule (stripping technique). Compared with drainage and coagulation of the endometrioma, excision has the advantage of providing material for histological diagnosis and excluding malignancy, and of having better results in relation to recurrence rate, dysmenorrhea, deep dyspareunia and non-menstrual pelvic pain.^(19)^

Endometrioma excision is associated with a considerable recurrence rate. In a study of 366 patients who underwent endometrioma excision, the following outcomes were observed after 48 months of follow-up: an US-diagnosed recurrence rate of 11.7%, a re-operation rate of 8.2%, and a pain recurrence rate of 73%.^(20)^

There is no scientific evidence for the use of hormonal medication before endometrioma surgery. Although such medications reduce the size of the endometrioma, they can induce capsular fibrosis and make surgery more difficult, thereby leading to greater loss of ovarian follicles.^(21)^

Studies examining the use of GnRH analogs following endometrioma surgery have demonstrated its ineffectiveness in preventing recurrence: both the group that used the medication and the non-user group showed an identical recurrence rate of 16.5% within 36 months.^(22)^

Studies are conflicting regarding the use of combined oral contraceptives after endometrioma surgery, but show lower recurrence in the group that underwent conservative surgery (stripping technique) and subsequently used contraceptives continuously or cyclically.^(23,24)^

## Endometrioma and risk of malignancy: myth or reality?

Although the chance of malignancy upon diagnosis of endometrioma is a concern, available studies indicate it is low, ranging from 0.8% to 0.9%. Case-control studies of ovarian cancer reveal a significantly increased risk (9.2% to 20.2%) of serous and low-grade endometrioid tumors in women with endometriosis. In this context, US evaluation may suggest the presence of signs of malignancy, as well as the follow-up by a gynecologic oncologist.^(25)^

## Serous or mucinous subtype

Ultrasound characteristics that indicate malignancy according to the International Ovarian Tumor Analysis (IOTA)^(25)^ include:

1. Irregular solid tumor;
2. Ascites;
3. At least four papillary structures;
4. Irregular multilocular solid tumor with the largest diameter ≥ 100 mm;
5. Increased blood flow on Doppler examination.

On the other hand, the five characteristics that suggest a mass is benign are:

1. Unilocular cyst;
2. Largest solid component < 7 mm;
3. Acoustic shadowing;
4. Regular multilocular tumor < 10 mm;
5. No detectable blood flow on Doppler examination.

The ESHRE recommends that women with endometriosis be informed that there is no significantly increased risk of cancer in general. Although endometriosis is associated with a slightly increased relative risk of developing ovarian, breast, and thyroid cancer, the absolute risk increase compared to the general population, is low.^(8)^

## Final considerations

The indications and techniques for the surgical approach to endometriomas still need to be established, so that we have well-defined procedures for each clinical situation. Asymptomatic endometriomas in older women should be treated to confirm the diagnosis, rule out malignancy in suspected cases, and prevent complications, mainly cyst rupture. The removal of asymptomatic endometriomas in women with a histological diagnosis of endometriosis is not advisable, as excision may decrease ovarian function. Laparoscopic excision of the endometrioma (stripping technique) is recommended, taking care to preserve normal ovarian tissue, instead of fenestration and ablation. The main criteria for performing surgical treatment include: intact ovarian reserve, no history of previous ovarian surgery, unilateral disease, rapid cyst growth, and uncertainty regarding the nature of the cyst. On the other hand, surgery should be avoided in cases with previous endometriosis surgery, reduced ovarian reserve, and bilateral endometriomas.

## Data Availability

the authors did not make the data for this article available in repositories prior to submission.

## National Specialized Commission on Endometriosis of the Brazilian Federation of Gynecology and Obstetrics Associations (FEBRASGO)

President:

Ricardo De Almeida Quintairos

Vice-president:

Marcia Mendonça Carneiro

Secretary:

Carlos Augusto Pires Costa Lino

Members:

Carlos Alberto Petta
Helizabeth Salomão Abdalla Ayroza Ribeiro
João Nogueira Neto
João Sabino Lahorgue Da Cunha Filho
Julio Cesar Rosa e Silva
Marcia Cristina Franca Ferreira
Marco Aurelio Pinho De Oliveira
Marcos Tcherniakovsky
Mauricio Simoes Abrao
Omero Benedicto Poli Neto
Raquel Papandreus Dibi
Sergio Podgaec
Sidney Pearce Furtado
